# Endoscopic retrograde appendicitis therapy in the management of chronic fecalith appendicitis in a patient with ulcerative colitis: The first human case report

**DOI:** 10.3389/fimmu.2022.1020393

**Published:** 2023-01-31

**Authors:** Muhammad Zulqarnain, Guangxing Cui, Wen Lyu, Haitao Huang, Xia Wang, Hamse Mohamoud Abdi, Lingfei Gu, Shijie Fang, Fangzhou Liu, Liqian Ling

**Affiliations:** ^1^ Department of Gastroenterology, Fourth Clinical Medical College of Zhejiang Chinese Medical University, Hangzhou, China; ^2^ Department of Gastroenterology, Affiliated Hangzhou First People’s Hospital, Zhejiang University School of Medicine, Hangzhou, Zhejiang, China

**Keywords:** chronic fecalith appendicitis, active ulcerative colitis, endoscopic retrograde appendicitis therapy (ERAT), antibiotics, inflammation

## Abstract

To assess the effectiveness of endoscopic retrograde appendicitis therapy (ERAT) as a new technique and method for chronic fecalith appendicitis complicated by active ulcerative colitis. A 46-year-old male patient was admitted with right iliac fossa pain, tenderness, and raised inflammatory markers. A computed tomography (CT) scan of his abdomen confirmed a dilated appendix, which is considered chronic fecalith appendicitis combined with active ulcerative colitis. He was treated with an endoscopic retrograde appendicitis therapy procedure. The patient recovered well after the ERAT procedure and was discharged from the hospital in two days. On follow-up one year later, there was no recurrence of pain in his abdomen. In conclusion, ERAT could be seen as a different approach and be favored as a safer and more effective option in treating UC patients with appendicitis, especially those who are later in the course of the disease. Because of the ERAT procedure, such cases can avoid surgery and surgery-related complications. More research and issues must be addressed to demonstrate the efficacy and effectiveness of ERAT in appendicitis combined with UC.

## Case presentation

**Figure 1:**
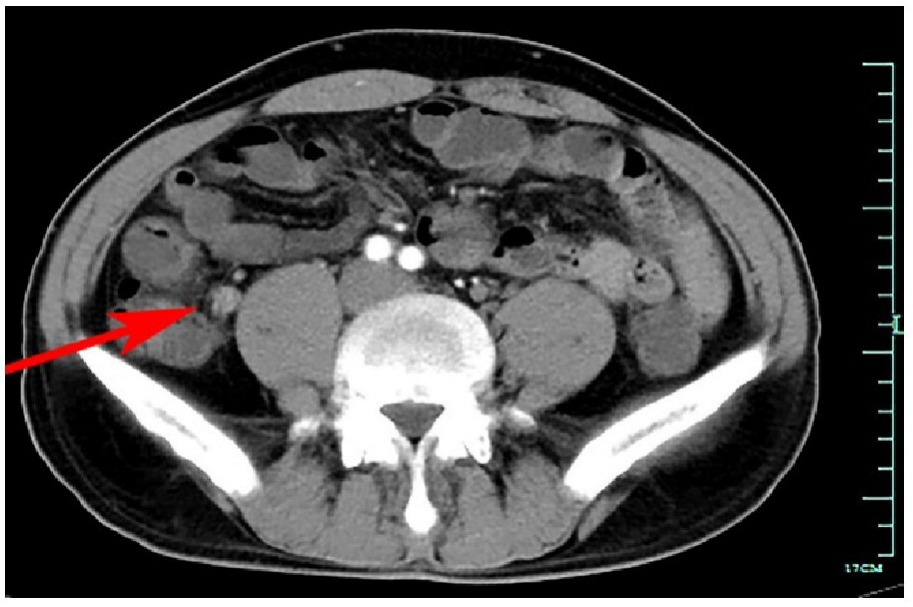
Abdominal contrast-enhanced computed tomography scan showing dilated appendix with high density in the lumen.

**Figure 2:**
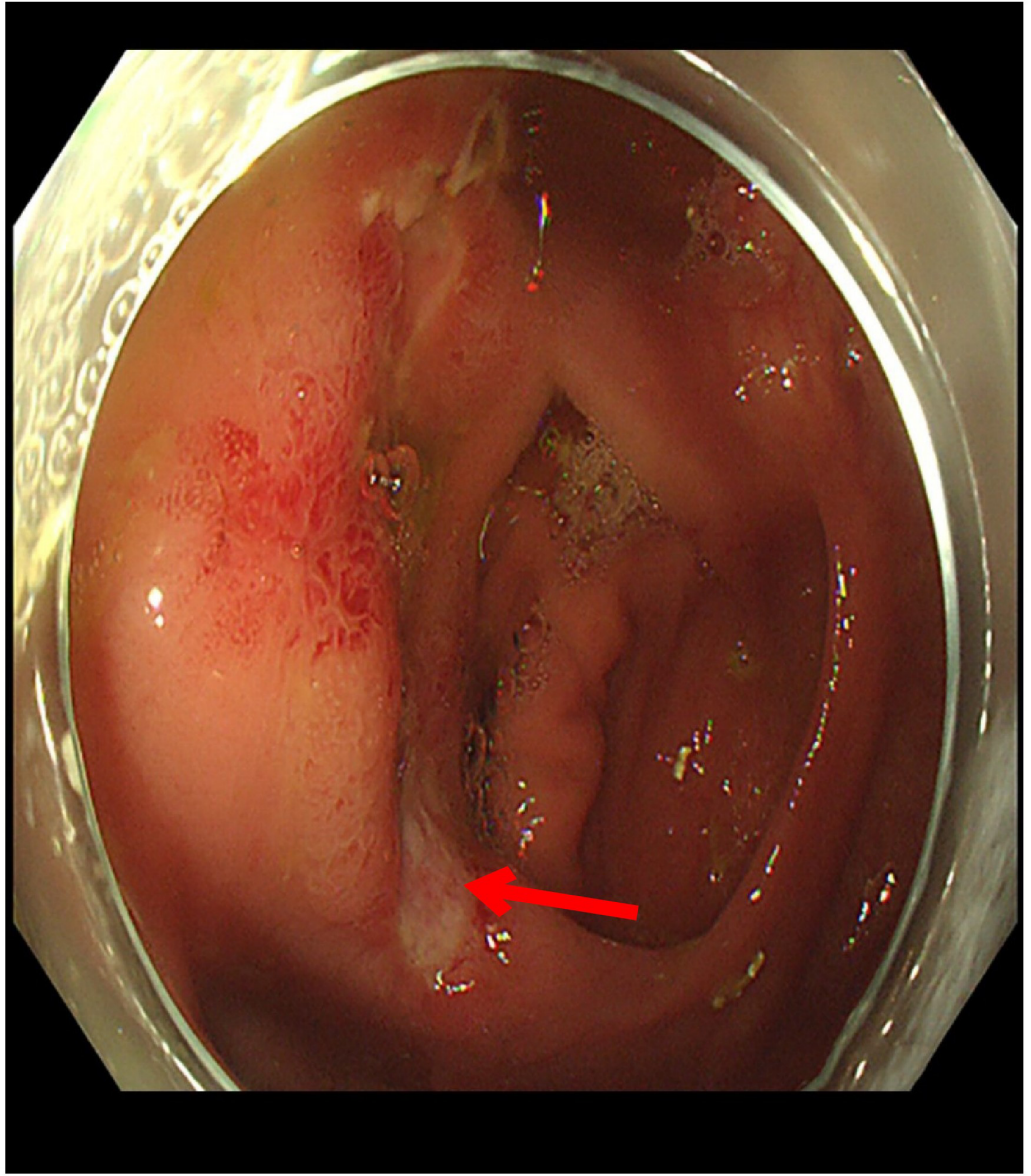
Large and irregular ulcer that is consistent with ulcerative colitis being seen on the ileocecal valve.

A 46-year-old Chinese male patient was admitted because of right lower abdominal pain for over two years that had been aggravated for one month. An abdominal CT scan revealed appendicitis and ulcerative colitis, which were suspected at a local hospital. As a result, the patient was referred to our hospital for further treatment. The patient denied a history of hypertension, coronary heart disease, cerebrovascular diseases, diabetes, and hyperthyroidism. During the physical examination, the temperature was 38.1°C, the abdomen was soft, there was positive tenderness, and there was no rebound pain. The auxiliary examination after admission showed that the white blood cells (WBC) were 13.8×109/L; hemoglobin was 111 g/L; C-reactive protein (CRP) was 90 mg/L (8 mg/L); the neutrophil percentage was 81.5%; and the procalcitonin was 0.55 ng/ml (0.5). Our hospital CT scan showed that the appendix was locally thickened and enhanced, chronic fecal appendicitis was suspected, and multiple small lymph nodes were displayed in the ileocecum area (**Figure 1**). Under colonoscopy, the terminal ileum and the orifice of the appendix were thickened, the ileocecal valve showed a deep ulcer, and the histopathological samples presented terminal ileum, ileocecal valve crypt abscess, and cryptitis. These findings most likely suggested chronic fecalith appendicitis with active ulcerative colitis. The final diagnosis was chronic fecal appendicitis with active ulcerative colitis. The ERAT procedure was performed for the patient. When the colonoscopy reached the ileocecal valve area, the mucosa appeared ulcerated, and the surface was covered with a crypt abscess, showing “ulcerative colitis”-like changes (**Figure 2**).

**Figure 3:**
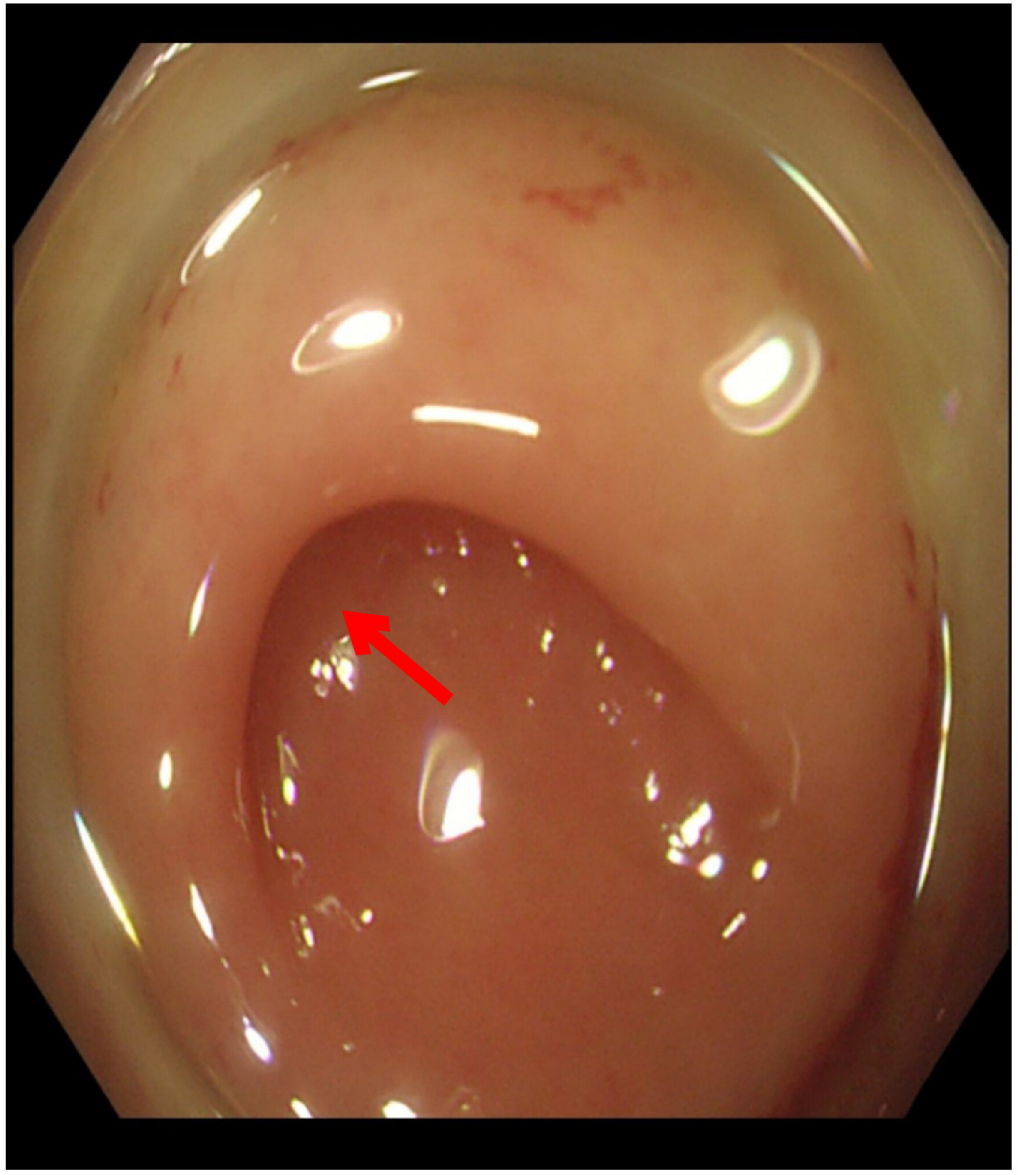
The appendiceal orifice is fully exposed with the aid of a transparent cap.

**Figure 4:**
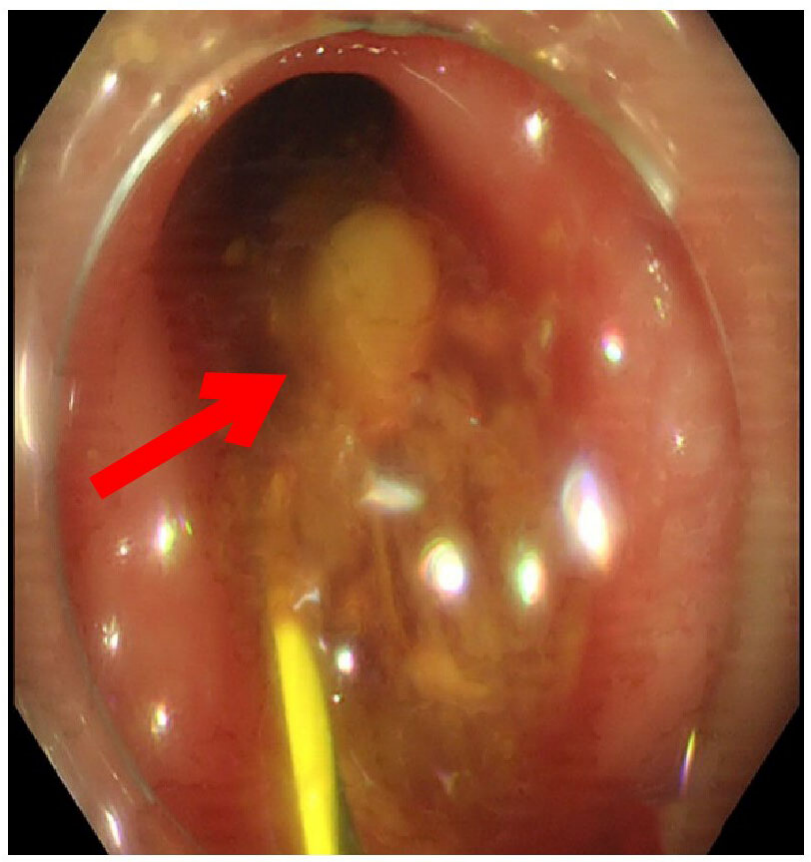
An appendiceal fecalith was flowing out during exchange of guidewire for balloon catheter, removal of fecalith with a balloon catheter.

**Figure 5:**
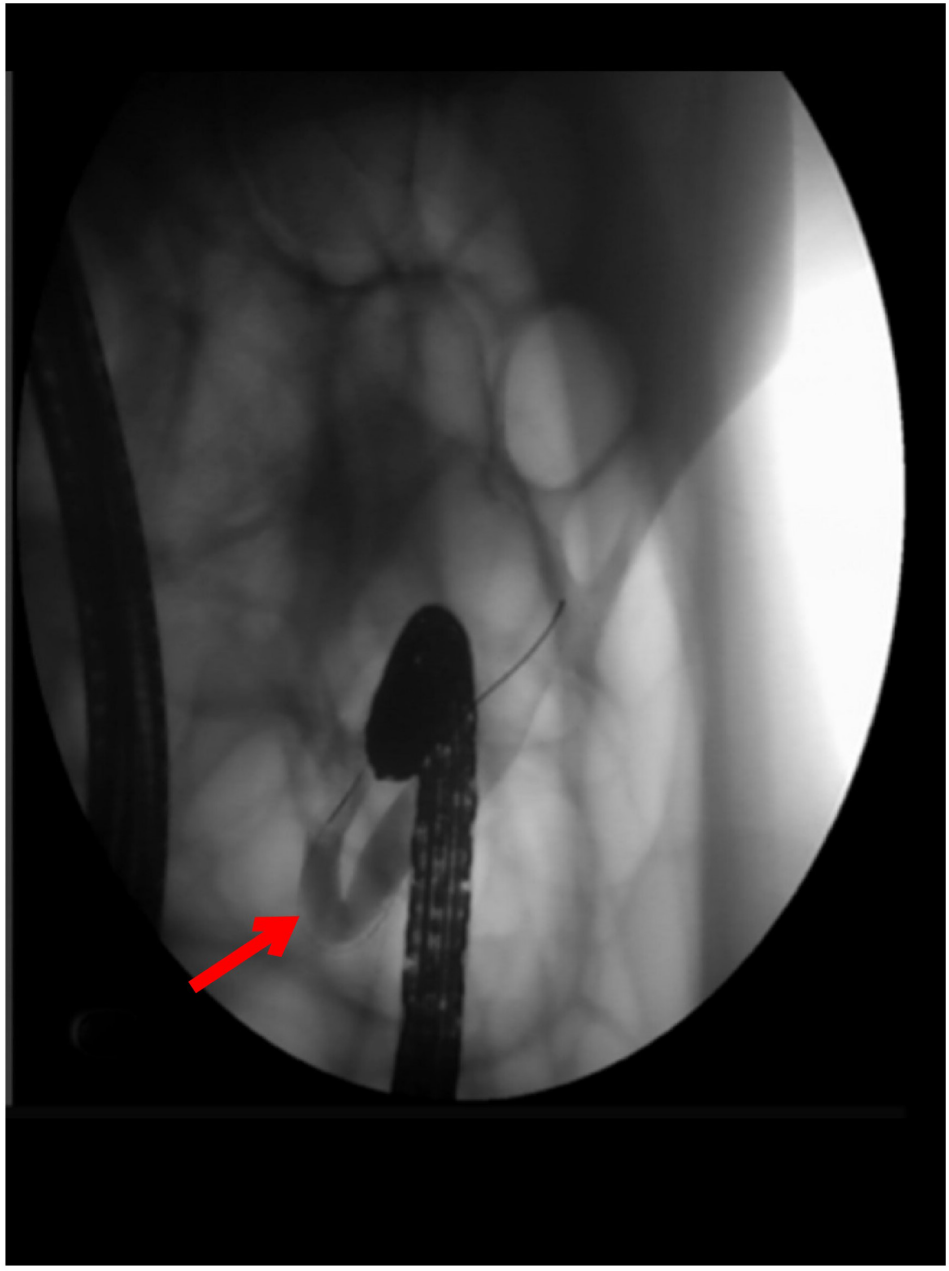
Fluoroscopy of the appendiceal lumen after successful cannulation showing the irregularly dilated appendiceal lumen with a diameter of 1.0cm and multiple filling defects.

**Figure 6:**
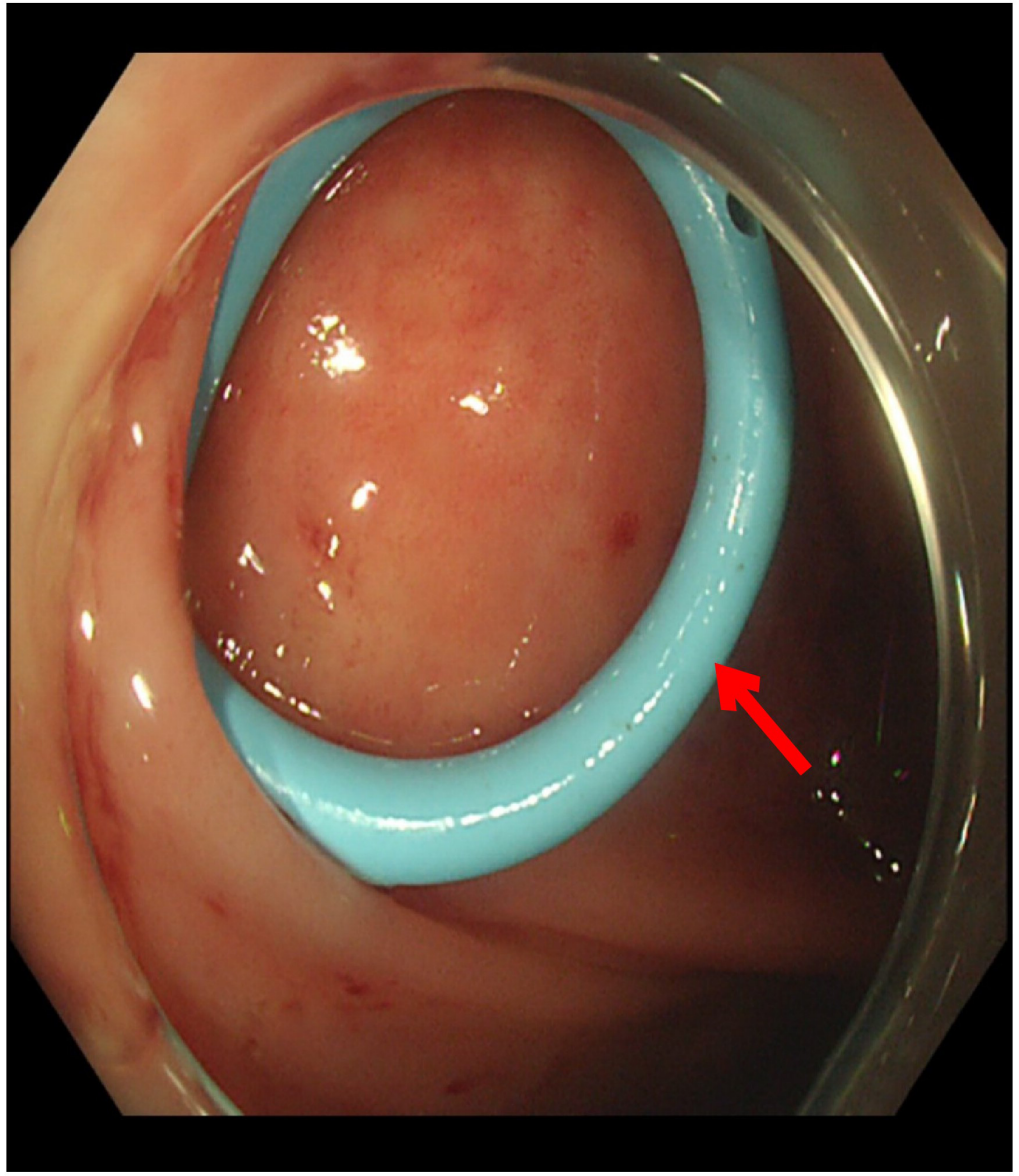
After clearance of the appendiceal lumen, a pigtail plastic stent with a size of 6 *French widths and 5 cm in length was successfully placed into the appendix cavity.

The orifice of the appendix was thickened, so it was challenging to insert the guide wire into the appendiceal orifice (**Figure 3**). Finally, we reached the appendiceal cavity, in which a copious amount of pus and stones could be seen flowing out during the procedure (**Figure 4**). Fluoroscopy revealed that the appendiceal lumen was severely dilated after successful cannulation, with a width of 10 mm, length of 8 cm, and an irregular shape (**Figure 5**). The appendiceal lumen was irrigated with 100 ml of metronidazole. After clearance of the appendiceal lumen, a pigtail plastic stent with a size of 6 French widths and 5 cm in length was successfully placed into the appendix cavity. (**Figure 6**). Intravenous antibiotics were administered before ERAT and two days after. The patient was discharged after the procedure, and the abdominal pain was relieved.

## Discussion

Ulcerative colitis (UC) is a chronic colon inflammation that has a variable clinical course (1). Although the etiology of UC is still unclear, genetic susceptibility and environmental factors are involved in the pathogenesis of UC. The results of a large, multi-center study demonstrate that appendectomy, regardless of the timing of the UC diagnosis, does not decrease the severity of the UC disease course compared to UC patients who did not undergo appendectomy. The results suggested that performing an appendectomy after a UC diagnosis may be harmful, as they showed a 2.2-fold increased risk of colectomy. An updated systematic review and meta-analysis confirmed the findings, which showed appendectomy did not affect colectomy rates in UC patients (2). Indications for surgery are intractability (49%), stricture, dysplasia, toxic colitis, bleeding, and perforation (3). Parian et al.’s retrospective database study of 2714 UC patients found a higher risk of colectomy in 48 patients who had appendectomy after UC diagnosis and concluded that appendectomy should not be recommended as a treatment for UC. However, recently published data show contradictory findings after appendectomy in UC patients, with an increased colectomy rate and, moreover, an increased risk of colorectal cancer (CRC) (4). If an appendectomy is associated with an increased risk of the subsequent development of CRC, this would have considerable implications for ongoing clinical studies and daily clinical practice. More research is necessary to understand this possible link and how the immune system might be involved. For over 100 years, appendectomy has been the first choice for treating acute appendicitis (5). It is now believed that the appendix has secretion and immune function (6, 7) and maintains the normal colonic flora (8). Appendectomy is not perfect because, overall, in-hospital complications of appendectomy occur as much as 11% (9). And the negative appendectomy rate remains as high as 8% (10). Endoscopic retrograde appendicitis therapy (ERAT) is an endoscopic therapy used to manage acute appendicitis (AA) and is an alternative to laparoscopic appendectomy. This method was first reported by Liu et al. in 2012. The recurrence rate of appendicitis following ERAT was low, with an average rate of 6.01 percent (11). The previous study reported a colonoscopic diagnosis of acute appendicitis and described the colonoscopic features of the appendiceal orifice during acute appendicitis (12–15). ERAT has several advantages. First of all, ERAT resulted in almost immediate relief from abdominal pain. Patients can resume normal activities after stent drainage and avoid postoperative pain from the incision. Second, ERAT is minimally invasive and allows patients with acute appendicitis to get treatment without a residual scar. ERAT is a simple and quick procedure that could be carried out in the outpatient department, thus further reducing medical expenses. Finally, ERAT retains the potential physiological function of the appendix. During the procedure, ulcerative colitis was seen in an operational condition, and inflammation was observed at the appendiceal orifice. Under fluoroscopy, the lumen was irregular and had no stricture. After the procedure, the patient’s abdominal pain disappeared. In addition to relieving the obstruction and removing Fecalith from the appendix lumen, draining the pus and flushing the lumen could control the inflammation. It also permits the insertion of a drainage tube into the lumen to ensure smooth drainage through the appendiceal orifice and reduce the risk of appendicitis recurrence caused by the blockage.

## Conclusion

ERAT is a different approach and is favored as a safer and more effective option in treating UC patients with appendicitis, especially those later in the course of the disease. Because of the ERAT procedure, such cases can avoid surgery and surgery-related complications. Follow-up one year later, the patient has no recurrent episodes of appendicitis. More research and issues must be addressed to demonstrate the efficacy and effectiveness of ERAT in appendicitis combined with UC.

## Data availability statement

The original contributions presented in the study are included in the article/supplementary material. Further inquiries can be directed to the corresponding author.

## Ethics statement

Written informed consent was obtained from the patient for publication of this case report and accompanying images.

## Author contributions

MZ and GC drafted the manuscript. GC and HH performed the ERAT procedure and designed the study. LG, SF, FL, LL and WX revised the manuscript. and HA is responsible for the collection of data and analysis. All authors contributed to the article and approved the submitted version.
